# Disproportionate cancer worries in ultra‐short‐segment Barrett's esophagus in Japan

**DOI:** 10.1002/deo2.329

**Published:** 2024-01-13

**Authors:** Sho Fukuda, Kenta Watanabe, So Takahashi, Tatsuki Yoshida, Shusei Fujimori, Taiga Komatsu, Yosuke Shimodaira, Tamotsu Matsuhashi, Katsunori Iijima

**Affiliations:** ^1^ Department of Gastroenterology Akita University Graduate School of Medicine Akita Japan; ^2^ Department of Gastroenterology Yokote Municipal Hospital Akita Japan; ^3^ Department of Anesthesiology Honjo‐Daiichi Hospital Akita Japan

**Keywords:** Barrett's esophagus, cancer perception, cancer risk, cancer worry, questionnaire survey

## Abstract

**Objectives:**

Although Barrett's esophagus (BE), especially ultra‐short‐segment BE (USSBE), is very frequently diagnosed in Japan, how subjects feel about receiving a diagnosis of BE is unclear. We therefore prospectively investigated cancer worry in subjects who received a BE diagnosis.

**Methods:**

Self‐administered questionnaires were sent to subjects who were diagnosed with BE at three health checkup institutes in Akita Prefecture, Japan. The cancer worry scale (CWS) was used to quantitatively assess the fear of developing cancer. The BE subjects were classified into USSBE <1 cm and non‐USSBE ≥1 cm groups. Factors associated with the CWS were investigated using logistic regression analyses.

**Results:**

A total of 325 (31%) subjects, comprising 229 USSBE and 96 non‐USSBE patients were included in this study. Compared with the USSBE group, the non‐USSBE group had a significantly higher frequency of a history of a BE diagnosis and perception of carcinogenesis. However, the CWS was similar between the USSBE and non‐USSBE groups, with a median CWS of 12.5 (3.75) versus 12.7 (3.65). A multivariate logistic regression analysis revealed that while positive reflux symptoms were significantly associated with a positive CWS, the BE length was not significantly associated with it, with an odds ratio (95% confidence interval) of 1.3 (0.75–2.2).

**Conclusions:**

A BE diagnosis promotes a similar level of worry about cancer among subjects, irrespective of the length of BE. In Japan, since USSBE poses a much lower cancer risk than non‐USSBE, the former may frequently be associated with a disproportionate cancer worry relative to the latter. (UMIN000044010)

## INTRODUCTION

The incidence of esophageal adenocarcinoma (EAC) has increased over the last several decades in Western countries,^1^ and recent reports have consistently indicated that it has begun to increase in Japan as well.^2^, ^3^ Barrett's esophagus (BE) is considered to be a premalignant condition of EAC, and in Europe and the United States, endoscopic surveillance is recommended for subjects with BE to detect EAC at an early stage.^4^ In the surveillance, the length of BE is considered the most important determinant for carcinogenic potential.^4^, ^5^ In Japan as well, there is growing interest in how to manage BE.^6^

The guidelines in Western countries have standardized the definition of BE by requiring at least a ≥ 1 cm length of columnar‐lined esophagus and/or histological confirmation of intestinal metaplasia.^7^ However, there is no such restriction in the definition of BE in Japan, where BE is generously diagnosed solely based on the endoscopic identification of columnar‐lined esophagus at any length without the need for a cumbersome histological confirmation.^7^, ^8^ Consequently, shorter forms of tiny BE are very frequently diagnosed, being detected in 15%–80% of operated endoscopic examinations in Japan,^9^ whereas this rate is only 5%–20% in Western countries.^10^ Given that the incidence of EAC is much lower in Japan at present than in Western countries,^11^ BE as a premalignant lesion for EAC is clearly overdiagnosed in Japan.

Aside from the potential for an over‐diagnosis of BE, such a generous diagnostic approach for BE in Japan lacks consideration from the patient's perspective,^12^, ^13^, ^14^ that is, it is unclear how subjects feel about receiving a diagnosis of BE and how they react to it. Cancer worry is defined as an emotional reaction to the threat of cancer,^15^ and it would be intriguing to investigate cancer worry among BE subjects in Japan, where BE is uniquely and frequently diagnosed. In the present study, we prospectively investigated cancer worry in subjects who received a BE diagnosis at a health checkup with special attention to the length of BE.

## METHODS

This quantitative, multicenter, self‐administered questionnaire study was recruited from three health checkup institutes serving esophagogastroduodenoscopy in Akita Prefecture, Japan, from May 2021 to March 2022. Consecutive subjects diagnosed with BE by endoscopy at the three institutes were potential candidates for this study. These subjects received an on‐site brief post‐endoscopy interaction with endoscopists or nurses. Several endoscopists were involved in the endoscopic examination at each institute. All medical staff at each institute were blinded to the study, and they treated the examinees as usual.

Approximately one month after the health checkup, an invitation letter to participate in this study was sent to the subjects who had been diagnosed with BE, together with the final notification report on the entire health checkup results in the usual format of each institute, including the endoscopic diagnosis of BE. The invitation letter included brief information on this study, an informed consent sheet, and the questionnaires described below. These subjects were instructed to complete the survey at home, and then send it to the research office at Akita University. The study protocol was approved by the ethics committee of Akita University and each participating institute (2620).

### Questionnaire

In addition to the following three standardized questionnaires, the subjects were asked about their perception of carcinogenesis with regard to BE (e.g., “Do you know that esophageal cancers can develop from BE?”), and the history of their BE diagnosis (e.g., “Have you ever been diagnosed with BE before?”), with “yes” or “no” as answers.

#### Cancer worry scale

The cancer worry scale (CWS) was originally developed to quantitatively assess concerns about cancer recurrence and the impact of these concerns on daily functioning in patients who had undergone treatment for cancers,^16^, ^17^ and more recently, it has been applied to assess the fear of developing cancers in those with precancerous conditions, such as familial adenomatous polyposis or BE.^18^, ^19^, ^20^, ^21^, ^22^ The scale comprises six‐item questions, with each rated on a 4‐point Likert scale ranging from 1 (“never”) to 4 (“almost always”).^18^, ^22^ Possible scores range from 6 to 24, with higher scores indicating more worry (Figure S1). In the present study, we used a Japanese‐translated version of the CWS, which was validated in a recent study.^23^

#### SF‐8

The health‐related quality of life was estimated using the SF‐8, which comprises 8 item questions.^24^

#### Frequency scale for the symptoms of gastro‐esophageal reflux disease

Gastro‐esophageal reflux disease (GERD)‐related upper gastrointestinal symptoms were evaluated using the frequency scale for the symptoms of GERD (FSSG), which is a widely used questionnaire in Japan.^25^ A FSSG score ≥8 indicates the presence of abnormal GERD‐related upper gastrointestinal symptoms.^25^

### Endoscopic findings

After completing a series of routine health checkup procedures, endoscopic images taken at the health checkup were reviewed independently by two investigators certified with the Japan Gastroenterological Endoscopy Society and blinded to the questionnaire results. Then, the investigators finally diagnosed the grade of BE (e.g. USSBE, <1 cm; short‐segment BE [SSBE], 1 to <3 cm; and long‐segment BE [LSBE], ≥3 cm). When there was disagreement concerning the diagnosis, a consensus was reached by joint review.

The gastro‐esophageal junction was defined as the anal side end of the palisade vessel or the oral side end of the fold continuous with the gastric lumen.^7^ BE was diagnosed in cases with columnar‐lined epithelium extending from the gastro‐esophageal junction to the esophagus of any length on endoscopy.^8^, ^26^

### Statistical analyses

Continuous and categorical variables were expressed as the mean (standard deviation [SD]) and the number (proportion) and compared using Student's *t*‐test and the chi‐square test, respectively. Ordinal scales were expressed as the median (interquartile range) and compared using the Mann‐Whitney U‐test. Because previous studies adopted a cut‐off of ≥12 as positive,^22^, ^27^ multivariate regression analyses were used to investigate factors associated with CWS using this cut‐off, and the results were expressed as the odds ratio (OR) and 95% confidence interval (CI).

All statistical analyses were conducted with the EZR software program (Saitama Medical Center, Jichi Medical University, Saitama, Japan),^28^ and *p*‐values of <0.05 were considered statistically significant.

## RESULTS

During this study period, a total of 4501 subjects received endoscopic examination at 3 health checkup institutes, and the 1045 (23.2%) diagnosed with BE were invited to participate in this study. On reviewing recorded endoscopic images, 758 (72.5%), 280 (26.8%), and seven (0.7%) patients were classified as having USSBE, SSBE, and LSBE, respectively. Consequently, 334 (32.0%) returned the questionnaire with informed consent. Subsequently, nine subjects were excluded from the analysis due to an incomplete questionnaire, leaving 325 (31.1%) as targets for the current study (Figure S2). Although the mean age was slightly higher among participants than non‐participants, the sex ratio and, importantly, the distribution of BE grading were similar between the two groups (Table S1). Since LSBE was diagnosed in only four target subjects, LSBE and SSBE were combined, and further analyses were done by classifying BE into two groups: 229 subjects with BE <1 cm (USSBE) and 96 with BE ≥1 cm (non‐USSBE; SSBE + LSBE).

Compared with USSBE, non‐USSBE had a significantly higher frequency of a history of a BE diagnosis (28.9% vs. 44.8%, *p* < 0.01) and perception of carcinogenesis (17.9% vs. 29.9%, *p* < 0.05; Table 1). Meanwhile, the median CWS was similar, irrespective of the grade of BE: 12.5 (3.75) in USSBE and 12.7 (3.65) in non‐USSBE. In addition, the prevalence of a positive CWS (≥12) was 62.0% in USSBE, which was not significantly different from that in non‐USSBE (70.8%). The distribution of institutes differed significantly between USSBE and non‐USSBE, indicating that the number of USSBE diagnoses relative to non‐USSBE diagnoses differed among participating institutes.

**TABLE 1:** Comparisons of investigated factors by the length of Barrett's esophagus.

Factors	The entire cohort (*n* = 325)	Length of Barrett's esophagus		
		<1 cm (*n* = 229)	≥1 cm (*n* = 96)	*p*‐value
Age, mean (SD)	58.4 (10.4)	58.5 (10.4)	57.9 (10.4)	0.65
Sex (male), *n* (%)	204 (62.8)	141 (61.6)	63 (65.6)	0.53
History of a BE diagnosis, *n* (%)	109 (33.5)	66 (28.9)	43 (44.8)	0.007
Perception of BE carcinogenesis, *n* (%)	69 (21.2)	41 (17.9)	28 (29.2)	0.026
FSSG, median (IQR)	6 (3,11)	5 (3, 11)	7 (3, 11)	0.168
SF‐8. MCS median (IQR)	51.7 (47.0, 54.6)	52.2 (47.0, 54.8)	51.3 (47.1, 54.6)	0.31
SF‐8. PCS median (IQR)	52.4 (47.9, 54.5)	51.8 (47.9, 53.9)	52.9 (47.9, 54.7)	0.38
Cancer Worry Scale, median (IQR)	12 (10,15)	12.5 (3.73)	12.7 (3.65)	0.55
Medication				
PPI, *n* (%)	59 (18.1)	39 (17.0)	20 (20.8)	0.43
Antidepressant drugs, *n* (%)	6 (1.8)	4 (1.7)	2 (2.1)	1
Endoscopic findings				
Hiatal Hernia, *n* (%)	43 (13.2)	19 (8.3)	24 (25.0)	< 0.001
Reflux esophagitis, *n* (%)	42 (12.9)	21 (9.2)	21 (21.9)	0.003
Institutes (N/H/Y), *n* (%)	34/143/148	24 (10.5)/120 (52.4)/85 (37.1)	10 (10.4)/ 23 (24.0)/ 63 (64.6)	<0.001

PPIs include potassium‐competitive acid blockers.

Abbreviations: BE, Barrett's esophagus; FSSG, frequency scale for the symptoms of gastro‐esophageal reflux disease; IQR, interquartile range; MCS, mental health component summary; PCS, physical health component summary; PPI, proton pump inhibitor; SD, standard deviation.

**FIGURE 1:**
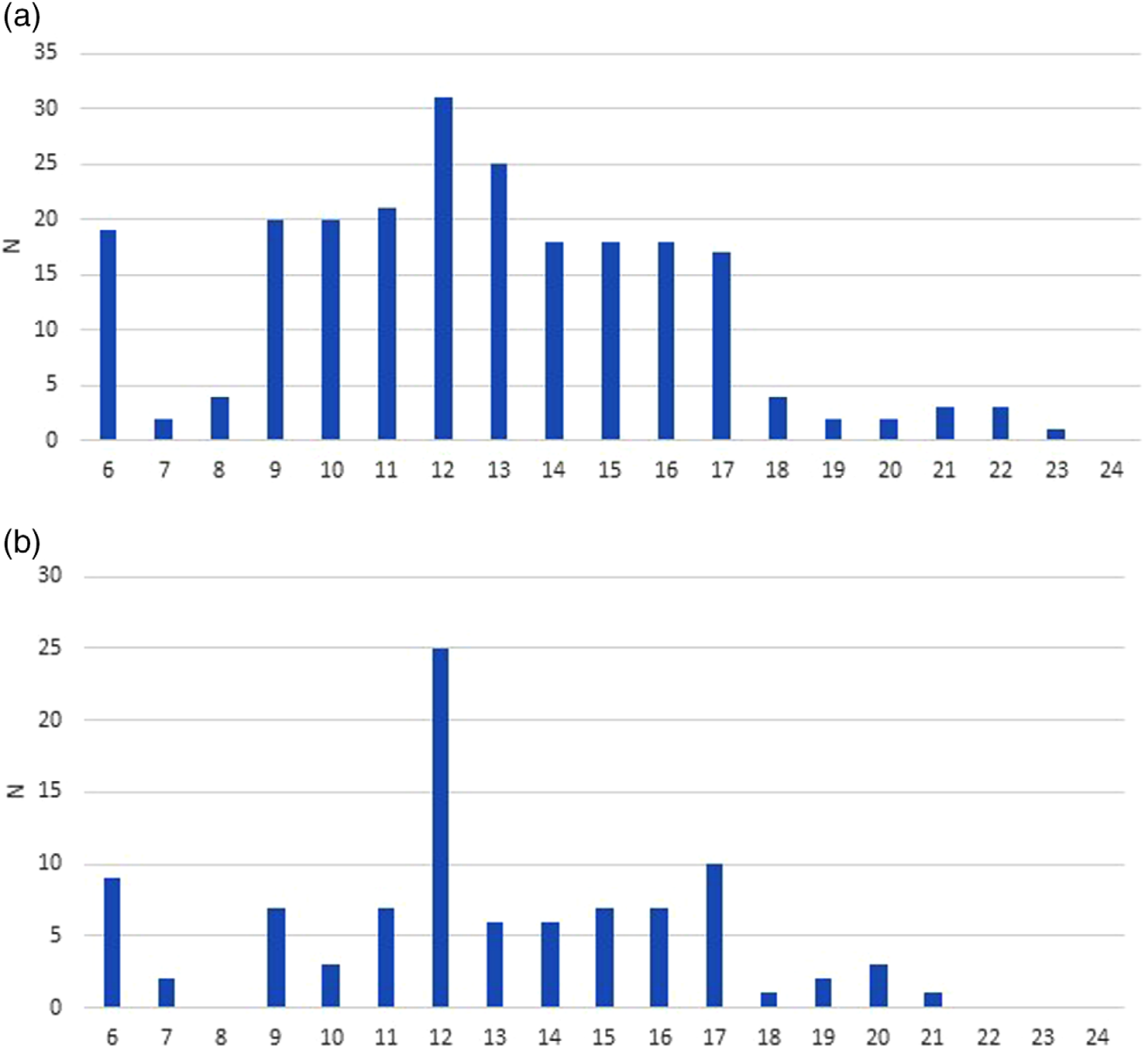
Histograms of cancer worry scales for esophageal cancer in those with ultra‐short‐segment Barrett's esophagus (<1 cm) (a) and non‐ultra‐short‐segment Barrett's esophagus (≥1 cm) (b).

The distribution pattern of the CWS was somewhat different between USSBE and non‐USSBE; while there was a single, steep peak in the CWS distribution in non‐USSBE, in USSBE, the CWS showed more widely scattered values around the median of 12 in USSBE (Figure 1).

**FIGURE 2:**
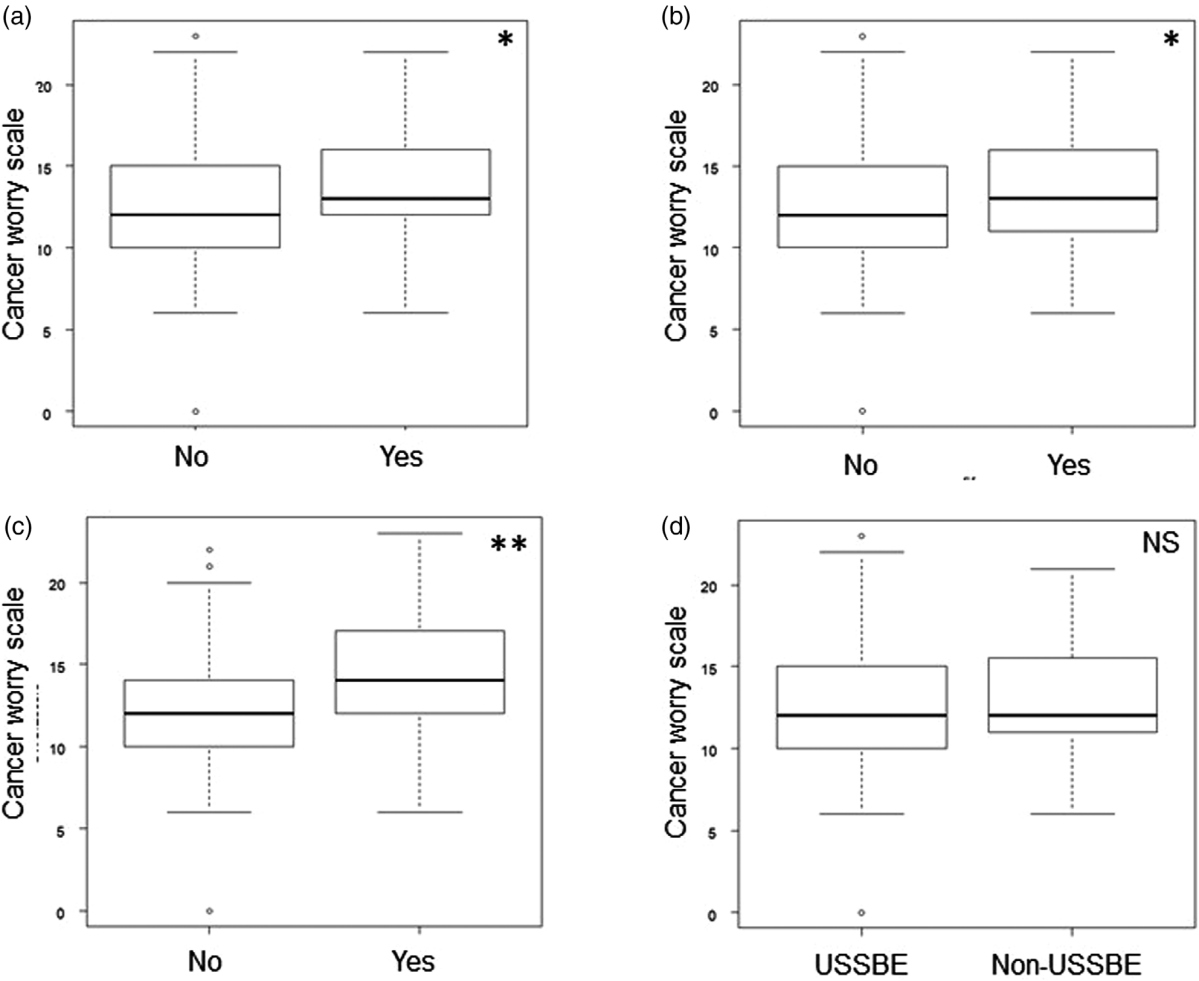
Comparisons of cancer worry scale for esophageal cancer by perception of carcinogenesis (a), the history of the diagnosis of Barrett's esophagus (b), positive gastro‐esophageal reflux symptoms (c), and the length of Barrett's esophagus (d). * or ** represents *p* < 0.05 or < 0.01, respectively. NS: not significant.

Two‐group comparisons of the CWS by various factors indicated that CWS was significantly higher in those with an understanding of BE carcinogenesis, a history of a BE diagnosis, positive GERD symptoms, and proton pump inhibitor intake than in others, although the values did not differ markedly by age, sex, or length of BE (Table S2 and Figure 2).

Univariate logistic regression analyses showed that an understanding of BE carcinogenesis and FSSG (≥8) was significantly associated with a positive CWS, defined as ≥12, with ORs (95% CIs) of 2.1 (1.1–3.9; *p* < 0.05) and 2.6 (1.5–4.2; *p* < 0.001), respectively. Furthermore, a multivariate logistic regression analysis revealed that FSSG (≥8) was the only independent risk factor for factor a positive CWS with OR (95% CI) of 2.4 (1.4–4.1; *p* < 0.01). Meanwhile, the length of BE was not significantly associated with the CWS in either univariate or multivariate analyses, with ORs (95% CIs) of 1.5 (0.9–2.5) and 1.3 (0.75–2.2), respectively (Table 2). As sensitivity analyses, logistic regression analyses were repeated using a positive CWS value, defined as ≥13 or ≥14, as an independent variable, and the outcomes were largely similar to those with the initial definition of ≥12 (Tables S3 and S4). Of note, the institute item was significantly associated with CWS when adopting a value of ≥14 as positive (Table S4).

**TABLE 2:** Logistic regression analyses for factors associated with positive cancer worry scale using a cut‐off of ≥12.

Factors	Univariate		Multivariate	
	OR (95% CI)	*p*‐value	OR (95% CI)	*p*‐value
Age, ≥75 years	0.85 (0.32–2.26)	0.71	0.80 (0.29–2.20)	0.51
Gender, male	0.9 (0.56–1.44)	0.66	1.06 (0.64–1.76)	0.77
Length of BE, non‐USSBE	1.49 (0.89–2.49)	0.13	1.29 (0.75–2.21)	0.21
FSSG, ≥8	2.55 (1.54–4.20)	< 0.001	2.41 (1.41–4.14)	0.0018
Perception of BE carcinogenesis, yes	2.09 (1.13–3.86)	0.019	1.46 (0.73–2.92)	0.27
History of a BE diagnosis, yes	1.61 (0.98–2.66)	0.059	1.36 (0.77–2.41)	0.25
SF‐8 MCS, ordinal	0.74 (0.5–1.11)	0.24	0.71 (0.42–1.22)	0.25
SF‐8 PCS, ordinal	1.25 (0.96–1.63)	0.10	1.05 (0.75–1.48)	0.64
PPI, yes	1.31 (0.71–2.40)	0.39	0.96 (0.49–1.86)	0.89
Institute, (ref: Y)				
H	1.15 (0.73–1.83)	0.54	0.54 (0.84–2.45)	0.19
N	1.0 (0.48–2.11)	0.99	1.5 (0.65–3.45)	0.33

PPIs include potassium‐competitive acid blockers.

Abbreviations: BE, Barrett's esophagus; CI, confidence interval; FSSG, frequency scale for the symptoms of gastro‐esophageal reflux disease; MCS, mental health component summary; OR, odd ratio; PCS, physical health component summary; PPI, proton pump inhibitor; USSBE, ultra‐short‐segment Barrett's esophagus.

Additional logistic regression analyses were performed to identify factors associated with the understanding of BE carcinogenesis. Univariate analysis showed that a history of a BE diagnosis, positive GERD symptoms, and a relatively long BE (non‐USSBE) were significantly associated with the understanding of BE carcinogenesis, and a BE diagnostic history and FSSG remained significant in the multivariate analysis. Noticeably, a history of a BE diagnosis was strongly associated with the understanding of BE carcinogenesis, with an OR (95% CI) of 9.6 (4.9–18.7; Table 3).

**TABLE 3:** Logistic regression analyses for factors associated with the perception of carcinogenesis of Barrett's esophagus.

Factors	Univariate		Multivariate	
	OR (95% CI)	*p*‐value	OR (95% CI)	*p*‐value
Age, ≥75 years	0.45 (0.1–2.0)	0.29	0.58 (0.11–3.09)	0.52
Gender, male	0.77 (0.45–1.33)	0.35	1.08 (0.56–2.06)	0.82
Length of BE, non‐USSBE	1.89 (1.08–3.29)	0.025	1.24 (0.64–2.47)	0.53
FSSG, ≥8	2.17 (1.27–3.72)	0.0048	2.43 (1.24–4.75)	0.0099
History of a BE diagnosis, yes	10.2 (5.5–19.1)	<0.001	9.58 (4.91–18.7)	<0.001
PPI, yes	1.34 (0.69–2.58)	0.385	1.15 (0.52–2.54)	0.73
SF‐8 MCS, ordinal	1.03 (0.65–1.64)	0.90	0.83 (0.40–1.74)	0.62
SF‐8 PCS, ordinal	0.91 (0.68–1.23)	0.55	0.80 (0.49–1.28)	0.34
Institute (ref: Y)				
H	0.97 (0.57–1.66)	0.92	1.00 (0.51–1.97)	0.99
N	0.21 (0.05–0.9)	0.035	0.56 (0.12–2.68)	0.47

PPIs include potassium‐competitive acid blockers.

Abbreviations: BE, Barrett's esophagus; CI, confidence interval; FSSG, frequency scale for the symptoms of gastro‐esophageal reflux disease; MCS, mental health component summary; OR, odds ratio; PCS, physical health component summary; PPI, proton pump inhibitor.

## DISCUSSION

This multicenter questionnaire survey revealed that cancer worry in those who had a diagnosis of BE is mainly determined by accompanying GERD symptoms, and importantly, that cancer worry is not determined by the subjects’ length of BE, a well‐recognized individual risk factor.^4^, ^5^

In Western countries, endoscopic surveillance targeting those with BE is widely recommended.^4^ In addition, some studies have investigated the psychological burden of being diagnosed with BE,^12^, ^13^, ^14^, ^17^, ^18^, ^19^, ^20^, ^21^ and demonstrated that while an appropriate amount of cancer worry was associated with increased motivation to undergo surveillance, excessive cancer worry resulting from an insufficient understanding of the disease or inaccurate perceptions of cancer risk may apply an unnecessary psychological burden on BE subjects.

In the current study, initially, 23.3% of subjects who received screening endoscopy at a health checkup were diagnosed with BE, most of whom (72.2%) had USSBE. Such a high prevalence of diagnosed BE is consistent with previous studies in Japan (e.g., 15%–80%).^8^ Then, among the participants of this study, 65% reported having substantial cancer worry defined as positive CWS ≥12 even just 1 month after a negative endoscopy for cancer at a health checkup. Importantly, cancer worry was similarly prevalent between USSBE and non‐USSBE, and a series of subsequent multivariate analyses consistently demonstrated that the length of BE was not associated with the CWS values. Thus, people may recognize a similar level of cancer worry from the diagnosis of BE itself, although the actual cancer risk is quite different depending on the subjects’ length of BE.^5^

Multivariate regression analyses consistently revealed that accompanying GERD symptoms are significantly associated with the degree of cancer worry in subjects with a BE diagnosis, which is consistent with those of recent studies from Western countries,^20^, ^21^ indicating that good control of symptoms can help reduce cancer worry in this cohort. In addition, at which institutes the subjects received the diagnosis may have made some impact on cancer worry among subjects (see Table S4). Therefore, in order to relieve cancer worry among subjects, it is important for medical teams at each institute to have good communication skills, particularly with regard to verbal reassurance about the diagnosis and the accompanying appropriate cancer risks when diagnosing patients with BE.^20^

An appropriate understanding of the cancer risk with BE is important to give BE patients an appropriate incentive to undergo surveillance.^19^, ^20^, ^21^ Previous studies demonstrated that having an appropriate perception of cancer risk does not necessarily lead to excessive cancer worry.^15^ This is supported by the findings of the current study, in which the understanding of BE carcinogenesis was only marginally associated with CWS. Interestingly, the current study also demonstrated that a history of a BE diagnosis is strongly associated with an understanding of BE carcinogenesis (e.g., OR of 9.6 in a multivariate analysis). Thus, giving constant and stable diagnoses of BE to applicable subjects will be useful for nurturing an appropriate understanding of BE carcinogenesis. Previous studies have consistently reported that the inter‐observer or inter‐institutional agreement for the diagnosis of USSBE is very poor, while that for non‐USSBE is at least moderate.^9^, ^29^ Thus, inconstant and unstable diagnoses frequently happen for USSBE, under which circumstances applicable subjects would fail to nurture an appropriate understanding of BE carcinogenesis and even ignore the diagnosis itself at worst, which may explain the scattered distribution of the CWS in USSBE patients in the present study (Figure 1).

One strength of this study is that employing a self‐administered questionnaire with signed informed consent instead of an anonymous questionnaire enabled us to collate the answer sheets to the clinical records of individual subjects, which was essential for classifying the applicable subjects into subgroups depending on the length of BE. In addition, to investigate the natural emotional response to the BE diagnosis at home, we employed postal invitation rather than on‐site recruitment in person. However, in exchange for its strength, the method inevitably led to a relatively low response rate (e.g., subjects would have felt more at ease answering an anonymous questionnaire), which may be one of the limitations of this study. Although a relatively low response rate was similarly reported in previous studies using postal invitations with signed questionnaires,^14^, ^19^ and the demographic data were largely similar between participants and non‐participants in this study, the low response rate to the questionnaire may suggest that the participants are more likely to be worried about cancer. Another limitation of this study is a lack of data on healthy controls. Thus, although we found similar levels of CWS between USSBE and non‐USSBE, the findings should be interpreted with caution. In addition, although the Japanese‐translated version of the CWS was validated in a recent study,^26^ the ideal cut‐off for a positive CWS value in Japanese people remains unclear. However, the results were largely similar even after adopting higher cut‐off values (e.g., ≥13 or ≥14) for a positive CWS. Furthermore, since only four subjects with LSBE participated in this analysis, we were unable to analyze the data specifically for LSBE. Since LSBE subjects possess a markedly higher cancer risk than those with shorter forms of BE,^29^, ^30^ it would be intriguing to investigate the CWS in this specific patient group. Further, we were unable to control subjects’ behavior at home after receiving a BE diagnosis in this study. Therefore, some subjects may have accessed available sources to gain information on BE, although this would usually happen in clinical settings as well. Finally, we did not employ a unified format in this study to inform subjects of BE diagnosis, instead, employed the usual format of each institute, because we would like to know the subjects’ emotional reaction to the notification report from the health check‐up. The presence or absence of BE alone was described without its grading in the format of each institute, which could affect the current outcomes (e.g., similar levels of CWS between USSBE and non‐USSBE). However, it would frequently happen in health check‐ups in Japan considering that cancer risks depending on the length of BE have not been clarified in Japan until very recently.^9^, ^30^, ^31^ An additional query on what explanation was given by the physicians would have been useful to measure its associations with patients’ anxiety.

In conclusion, this is the first study to investigate how individuals emotionally reacted to receiving a diagnosis of BE in Japan, with findings showing that the diagnosis gave a similar level of cancer worry to subjects regardless of the individual length of BE, a well‐established risk factor for cancer.^4^, ^5^ In Japan, since USSBE possesses a much lower cancer risk than longer forms of BE,^9^, ^30^, ^31^ a USSBE diagnosis may apply a disproportionate degree of cancer worry relative to non‐USSBE. Endoscopists and endoscopy centers should therefore take care to not only perform a rigorous diagnosis of BE but also be mindful of patients’ anxiety about their diagnosis. When endoscopists give a diagnosis of USSBE to patients, it should be done by adding comments that it has a very low cancer risk, or more simply, diagnosing only columnar‐lined esophagus ≥1 cm as BE, as in some guidelines in Western countries,^7^ may be another practical solution to avoid distress to the patients.

## CONFLICT OF INTEREST STATEMENT

Katsunori Iijima received lecture fees from Takeda Pharmaceutical Co., Ltd. and Otsuka Pharmaceutical Co., Ltd.

## Supporting information

Supplemental Table 1: Comparisons of demographic factors between participants and non‐participants to the study.
Supplemental Table 2: Comparisons of cancer worry scale by investigated factors.
Supplemental Table 3: Logistic regression analyses for factors associated with positive cancer worry scale using a cut‐off of ≥13.
Supplemental Table 4: Logistic regression analyses for factors associated with positive cancer worry scale using a cut‐off of ≥14

Figure S1 Items of cancer worry scale

Figure S2 Flowchart of the subjects participating in this study
