# Supplementary material for: Disproportionate cancer worries in ultra‐short‐segment Barrett's esophagus in Japan
Source: DEN Open. 2024 Jan 13;4(1):e329. doi: 10.1002/deo2.329 (PMC10787273; doi:10.1002/deo2.329)
Supplement: Supplementary file 1 — Supplemental Table 1: Comparisons of demographic factors between participants and non‐participants to the study. Supplemental Table 2: Comparisons of cancer worry scale by investigated factors. Supplemental Table 3: Logistic regression analyses for factors associated with positive cancer worry scale using a cut‐off of ≥13. Supplemental Table 4: Logistic regression analyses for factors associated with positive cancer worry scale using a cut‐off of ≥14 [file DEO2-4-e329-s002.docx]

**Supplemental Table 1: Comparisons of demographic factors between participants and non-participants to the study**

|  | **Participants**  **n=325 (31.1%)** | **Non-participants**  **n=720 (68.9%)** | **P value** |
| --- | --- | --- | --- |
| Age, years; mean (SD) | 58.4 (10.4) | 56.9 (10.4) | 0.049 |
| Sex (male), n (%) | 204 (62.8) | 473 (65.7) | 0.44 |
| Length of BE  (USSBE/SSBE/LSBE), n (%) | 229 (70.5) / 93 (28.6) /3 (0.9) | 529 (73.5) / 187 (26) / 4 (0.5) | 0.32 |

BE: Barrett’s esophagus; USSBE: ultra-short-segment Barrett’s esophagus, SSBE: short-segment Barrett’s esophagus, LSBE: long-segment Barrett’s esophagus

**Supplemental Table 2: Comparisons of cancer worry scale by investigated factors**

| **Factors** | **Subgroups** | **Cancer Worry Scale, median (IQR)** | **P value** |
| --- | --- | --- | --- |
| Gender | Male | 12 (12, 15) | 0.28 |
|  | Female | 12 (10, 16) |  |
| Age | ≥ 75 | 12 (10.3, 16.8) | 0.80 |
|  | <75 | 12 (10, 15) |  |
| Perception of BE carcinogenesis | Yes | 13 (12, 16) | 0.011 |
|  | No | 12 (10, 15) |  |
| History of a BE diagnosis | Yes | 13 (11, 16) | 0.025 |
|  | No | 12 (10, 15) |  |
| FSSG | ≥8 | 14 (12, 16.8) | <0.0001 |
|  | <8 | 12 (10, 14) |  |
| PPI | Yes | 13 (11, 17) | 0.036 |
|  | No | 12 (10, 15) |  |
| Length of BE | USSBE | 12 (11, 15.3) | 0. 55 |
|  | Non-USSBE | 12 (10, 15) |  |
| Institutes | N | 13 (10, 15.8) | 0.50 |
|  | H | 13 (10, 15.5) |  |
|  | Y | 12 (10, 15) |  |

PPIs include potassium-competitive acid blockers.

BE: Barrett’s esophagus, FSSG: frequency scale for the symptoms of GERD, PPI: proton pump inhibitor, USSBE: ultra-short-segment Barrett’s esophagus, IQR: interquartile range

**Supplemental Table 3: Logistic regression analyses for factors associated with positive cancer worry scale using a cut-off of ≥13**

| **Factors** | **Univariate** | |  | **Multivariate** | |
| --- | --- | --- | --- | --- | --- |
|  | **OR (95% CI)** | **P value** |  | **OR (95% CI)** | **P value** |
| Age, ≥75 years | 0.69 (0.26-1.83) | 0.46 |  | 0.57 (0.20-1.61) | 0.29 |
| Gender, male | 0.87 (0.55-1.39) | 0.54 |  | 1.04 (0.64-1.7) | 0.88 |
| Length of BE, non-USSBE | 0.86 (055-1.39) | 0.54 |  | 0.78 (0.45-1.33) | 0.36 |
| FSSG, ≥8 | 2.49 (1.58-3.94) | < 0.001 |  | 2.25 (1.36-3.73) | 0.0017 |
| Perception of BE carcinogenesis, yes | 1.85 (1.08-3.18) | 0.025 |  | 1.4 (0.74-2.63) | 0.30 |
| History of a BE diagnosis, yes | 1.52 (0.96-2.41) | 0.077 |  | 1.58 (0.91-2.78) | 0.11 |
| SF-8 MCS, ordinal | 1.21 (0.95-1.54) | 0.12 |  | 0.94 (0.55-1.59) | 0.81 |
| SF-8 PCS, ordinal | 0.88 (0.6-1.29) | 0.51 |  | 1.14 (0.81-1.59) | 0.45 |
| PPI, yes | 1.8 (1.02-1.92) | 0.044 |  | 1.54 (0.82-2.87) | 0.18 |
| Institute (ref: Y) |  |  |  |  |  |
| H | 1.16 (0.74-1.83) | 0.51 |  | 0.54 (0.84-2.45) | 0.19 |
| N | 1.0 (0.48-2.11) | 0.99 |  | 1.5 (0.65-3.45) | 0.33 |

PPIs include potassium-competitive acid blockers.

BE: Barrett’s esophagus, USSBE: ultra-short-segment Barrett’s esophagus, FSSG: frequency scale for the symptoms of GERD, PCS: physical health component summary, MCS: mental health component summary, PPI: proton pump inhibitor, OR: odd ratio, CI: confidence interval

**Supplemental Table 4: Logistic regression analyses for factors associated with positive cancer worry scale using a cut-off of ≥14**

| **Factors** | **Univariate** | |  | **Multivariate** | |
| --- | --- | --- | --- | --- | --- |
|  | **OR (95% CI)** | **P value** |  | **OR (95% CI)** | **P value** |
| Age, ≥75 years | 1.05 (0.39-2.78) | 0.92 |  | 0.9 (0.31-2.55) | 0.84 |
| Gender, male | 0.67 (0.42-1.06) | 0.089 |  | 0.78 (0.47-1.28) | 0.32 |
| Length of BE, non-USSBE | 1.04 (0.64-1.7) | 0.87 |  | 0.97 (0.56-1.68) | 0.92 |
| FSSG, ≥8 | 2.59 (1.63-4.12) | < 0.001 |  | 2.36 (1.42-3.94) | <0.001 |
| Perception of BE carcinogenesis, yes | 1.45 (0.85-2.49) | 0.17 |  | 1.05 (0.55-2.01) | 0.88 |
| History of a BE diagnosis, yes | 1.56 (0.97-2.49) | 0.065 |  | 1.74 (0.98-3.1) | 0.059 |
| SF-8 MCS, ordinal | 0.83 (0.56-1.23) | 0.36 |  | 0.95 (0.55-1.63) | 0.85 |
| SF-8 PCS, ordinal | 1.26 (0.98-1.61) | 0.067 |  | 1.19 (0.84-1.67) | 0.33 |
| PPI, yes | 1.49 (0.85-2.64) | 0.17 |  | 1.24 (0.66-2.32 | 0.50 |
| Institute (ref: Y) |  |  |  |  |  |
| H | 1.16 (0.74-1.83) | 0.51 |  | 0.46 (0.86-2.49) | 0.16 |
| N | 1.51 (0.75-3.12) | 0.24 |  | 2.32 (1.1-5.76) | 0.028 |

PPIs include potassium-competitive acid blockers.

BE: Barrett’s esophagus, USSBE: ultra-short-segment Barrett’s esophagus, FSSG: frequency scale for the symptoms of GERD, PCS: physical health component summary, MCS: mental health component summary, PPI: proton pump inhibitor, OR: odd ratio, CI: confidence interval
