# Supplementary figures and images for: Disproportionate cancer worries in ultra‐short‐segment Barrett's esophagus in Japan
Source: DEN Open. 2024 Jan 13;4(1):e329. doi: 10.1002/deo2.329 (PMC10787273; doi:10.1002/deo2.329)

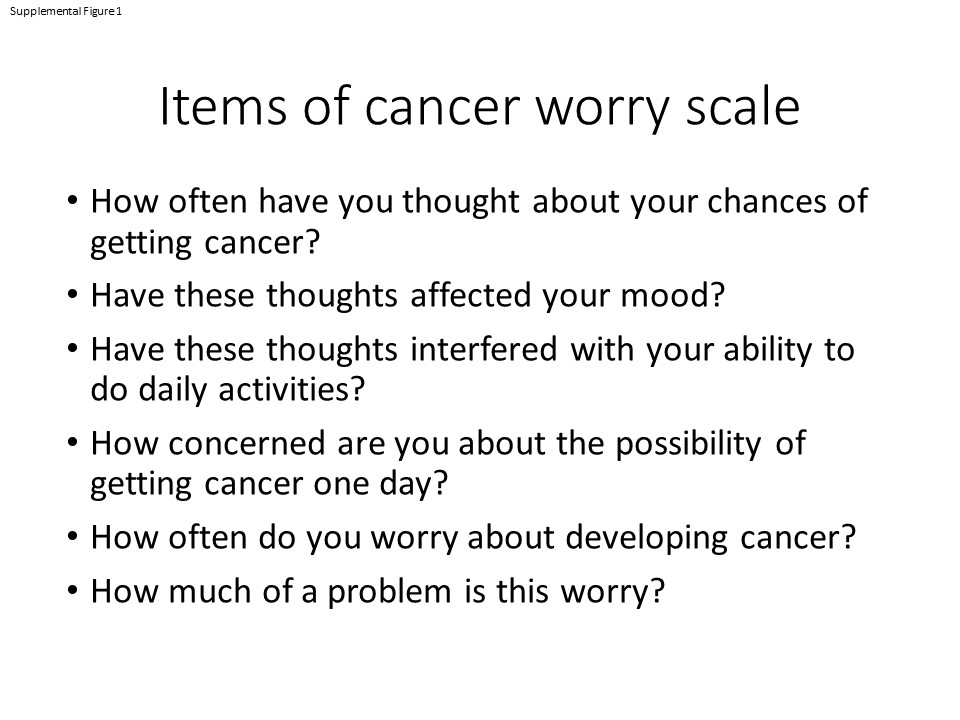

Supplement: Supplementary file 2 — Figure S1 Items of cancer worry scale [file DEO2-4-e329-s003.JPG]

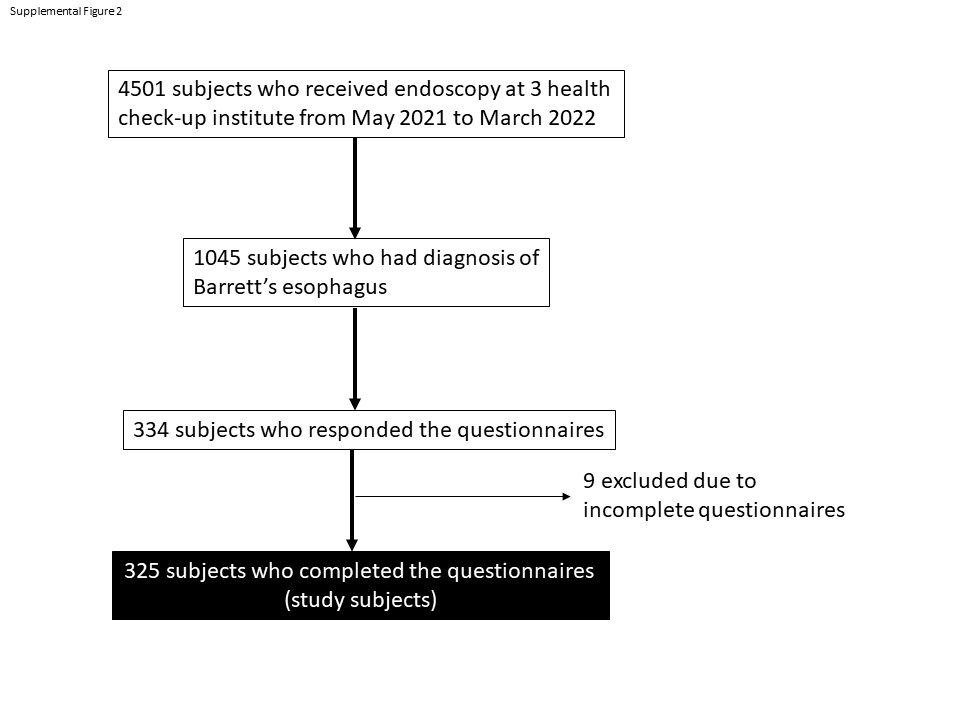

Supplement: Supplementary file 3 — Figure S2 Flowchart of the subjects participating in this study [file DEO2-4-e329-s001.JPG]
